# Supplementary material for: Tuning magnetoelectricity in a mixed-anisotropy antiferromagnet
Source: Nat Commun. 2023 Jun 9;14:3408. doi: 10.1038/s41467-023-39128-7 (PMC10256750; doi:10.1038/s41467-023-39128-7)
Supplement: Supplementary file 1 — Supplementary Information [file 41467_2023_39128_MOESM1_ESM.pdf]

Supplementary information for

Tuning magnetoelectricity in a mixed-anisotropy antiferromagnet

Ellen Fogh, Bastian Klemke, Manfred Reehuis, Philippe Bourges, Christof Niedermayer, Sonja Holm-Dahlin, Oksana Zaharko, Jürg Schefer, Andreas B. Kristensen, Michael K. Sørensen, Sebastian Paeckel, Kasper S. Pedersen, Rasmus E. Hansen, Alexandre Pages, Kimmie K. Moerner, Giulia Meucci, Jian-Rui Soh, Alessandro Bombardi, David Vaknin, Henrik M. Rønnow, Olav F. Syljuåsen. Niels B. Christensen and Rasmus Toft-Petersen

Neutron diffraction experiments

In the crystal structure of LiMPO$_4$, the transition metal ions reside on the $4c$ Wyckoff positions with coordinates $r_1 = (1+\varepsilon, 1/4, 1-\delta)$, $r_2 = (3/4+\varepsilon, 1/4, 1/2+\delta)$, $r_3 = (3/4-\varepsilon, 3/4, \delta)$ and $r_4 = (1/4-\varepsilon, 3/4, 1/2-\delta)$, with $\varepsilon = 0.025$, $\delta = 0.0175$ for LiNiPO$_4$ [1] and $\varepsilon = 0.032$, $\delta = 0.0252$ for LiFePO$_4$ [2]. To analyse the magnetic structures with propagation vector $\mathbf{k} = (0,0,0)$ that are experimentally found in LiMPO$_4$ [3? , 4], we consider four basis vectors corresponding to the specific sequences of spins $A_\alpha = (\uparrow\downarrow\downarrow\uparrow)$, $G_\alpha = (\uparrow\downarrow\uparrow\downarrow)$, $C_\alpha = (\uparrow\uparrow\downarrow\downarrow)$ and $F_\alpha = (\uparrow\uparrow\uparrow\uparrow)$ on the sites r_1-r_4. When combining these four basis vectors with three orthogonal spin directions, α, the resulting 12 components allow a full description of the possible magnetic structures.

For unpolarized neutrons the differential scattering cross-section corresponding to each component of the magnetic structure is proportional to $|S_R(\mathbf{Q})|^2 |\mathcal{P}_\mathbf{e}(\mathbf{Q})|^2$, where

$$S_R(\mathbf{Q}) = \sum_d m_d^R e^{i\mathbf{Q}\cdot\mathbf{r}_d}, \tag{1}$$

$$\mathcal{P}_\mathbf{e}(\mathbf{Q}) = \hat{\mathbf{Q}} \times (\hat{\mathbf{e}} \times \hat{\mathbf{Q}}). \tag{2}$$

Here $S_R(\mathbf{Q})$ is a structure factor involving the summation over the magnetic moments of the ions at sites r_1-r_4 and R refers to the particular basis vector of the magnetic structure. The vector $\hat{\mathbf{Q}}$ is a unit vector along the neutron momentum transfer in the scattering process, and $\hat{\mathbf{e}}$ is a unit vector along the direction of the magnetic moments for the component R. The factor $\mathcal{P}_\mathbf{e}(\mathbf{Q})$ reflects the fact that neutrons only scatter from electronic spin components perpendicular to $\mathbf{Q}$.

Table I lists $|S_R(\mathbf{Q})|^\mathbf{2}$ and $|\mathcal{P}_\mathbf{e}(\mathbf{Q})|^\mathbf{2}$ for the basis vectors C, A, G, F and spin directions $\hat{\mathbf{e}}$ parallel to a, b or c for the Bragg peaks probed in our experiments. The entries of the table represent the sensitivity of each magnetic Bragg peak to the different basis structure components. For example, the peaks $(0,0,\pm1)$ are dominated by components corresponding to the basis vector G_a and G_b with minor contributions from components C_a and C_b. On the other hand, observing $(0,1,0)$ implies that at least one of the components C_a and C_c must be present. If the magnetic structure were exclusively described by the C_y component, the $(0,1,0)$ peak would have no intensity because the magnetic moments are parallel to $\mathbf{Q}$ and from which follows $\mathcal{P}_b(\mathbf{Q}) = 0$.

For our experiments using uniaxial polarization analysis, the relevant expressions for the non spin-flip (NSF) scattering intensities are

$$\begin{aligned} I_x^{\rm NSF} &= I_{\rm coh}^N + B_{\rm NSF}, & (3)\\ I_y^{\rm NSF} &= I_{\rm coh}^N + I_y^M + B_{\rm NSF}, & (4)\\ I_z^{\rm NSF} &= I_{\rm coh}^N + I_z^M + B_{\rm NSF}, & (5)\end{aligned}$$

while those for spin-flip (SF) intensities are

$$\begin{aligned} I_x^{\rm SF} &= I_y^M + I_z^M + B_{\rm SF}, & (6)\\ I_y^{\rm SF} &= I_z^M + B_{\rm SF}, & (7)\\ I_z^{\rm SF} &= I_y^M + B_{\rm SF}. & (8)\end{aligned}$$

These expressions are valid when contributions from nuclear-magnetic interference scattering and chiral scattering can be neglected [5]. The subscripts x, y and z denote the direction of neutron polarization where x corresponds to the configuration where the neutron polarization is parallel to $\mathbf{Q}$, y is when the polarization is perpendicular to $\mathbf{Q}$ and in the horizontal scattering plane, while z refers to when the polarization is perpendicular to the scattering plane. The coordinate system is illustrated in Fig. 2c. The contribution $I_{\rm coh}^N$ to the three NSF cross-sections corresponds to nuclear Bragg scattering. The terms I_y^M and I_z^M are magnetic contributions to a given Bragg peak intensity, arising from electronic spin components parallel to y and z, respectively. Note that the NSF contributions carry information on spin components along the neutron polarization, while the SF contributions carry information on spin components perpendicular to the neutron polarization. The background contribution $B_{\rm NSF}$ in the NSF channels involves the sum of nuclear isotope incoherent scattering and one third of the total nuclear spin incoherent scattering. The background term $B_{\rm SF}$ in the SF channels involves the remaining two thirds of the nuclear spin incoherent scattering.

Because the polarization of the neutron beam is never perfect, a correction is needed before the measured Bragg peak intensities can be directly compared to the above expressions for the polarized neutron diffraction intensity. The relevant correction factors, also called flipping ratios, were determined from measurements of nuclear Bragg peaks above T_2. We found flipping ratios 40 (25) for nuclear scattering vectors along the b^* (c^*) direction, respectively. For more details on the analysis see e.g. Refs. [6], [5] or [7].

TABLE I. Structure factors for various magnetic Bragg peaks as composed by the four basis vectors $A = (\uparrow\downarrow\downarrow\uparrow)$, $G = (\uparrow\downarrow\uparrow\downarrow)$, $C = (\uparrow\uparrow\downarrow\downarrow)$ and $F = (\uparrow\uparrow\uparrow\uparrow)$. The last three columns contain the factors $|\mathcal{P}_\alpha(\mathbf{Q})|^2$ for the three crystallographic axes, $\alpha = \{a, b, c\}$. The factors are normalized to unit spin length.

$\mathbf{Q}$	$\lvert S_A(\mathbf{Q})\rvert^2$	$\lvert S_G(\mathbf{Q})\rvert^2$	$\lvert S_C(\mathbf{Q})\rvert^2$	$\lvert S_F(\mathbf{Q})\rvert^2$	$\lvert\mathcal{P}_a(\mathbf{Q})\rvert^2$	$\lvert\mathcal{P}_b(\mathbf{Q})\rvert^2$	$\lvert\mathcal{P}_c(\mathbf{Q})\rvert^2$
$(0,0,\pm1)$	0	15.05	0.50	0	1	1	0
$(0,1,0)$	0	0	16	0	1	0	1
$(3,0,-1)$	0.16	0.35	10.60	4.91	0.34	1	0.66
$(0,1,-2)$	0	1.93	14.07	0	1	0.86	0.14
$(1,0,0)$	15.37	0.63	0	0	0	1	1
$(1,1,0)$	0.63	15.37	0	0	0.75	0.25	1
$(1,2,0)$	15.37	0.63	0	0	0.92	0.08	1
$(2,1,0)$	0	0	13.59	2.41	0.41	0.58	1

E5 experiment

The room temperature crystal structure of $LiNi_{0.8}Fe_{0.2}PO_4$ was determined from 3255 reflections collected with $\lambda = 0.896$ Å and from which 724 are unique. The refinement of a total of 41 parameters was carried out assuming space group $Pnma$ and gives a residual of $R_F = \sum \frac{F_{\rm obs}-F_{\rm calc}}{F_{\rm obs}} = 0.050$ where $F_{\rm obs}$ and $F_{\rm calc}$ are the observed and calculated intensities, respectively. We used the following lattice parameters as obtained from UB matrix calculations using strong reflections during the experiment setup: $a = 9.9741(14)$ Å, $b = 5.8284(6)$ Å, $c = 4.6326(5)$ Å. The refinement results are summarized in Table II. The ion displacements away from a perfect face centered configuration are $\varepsilon = 0.02701(5)$ and $\delta = 0.01877(10)$, values which not surprisingly lie in between those of the parent compounds.

The temperature dependency of the neutron diffraction intensity of 8 different magnetic Bragg peaks measured with $\lambda = 2.38$ Å is shown in Fig. 1. Some peaks, such as $(0,1,0)$, display a power law behavior with onset at around 21 K. Other peaks, like $(0,0,-1)$, display a linear temperature dependence with an onset temperature close to 25 K. Finally, peaks such as $(3,0,-1)$, display both trends. All peak intensities were fitted to the following function:

$$I(T) = \begin{cases} B & \text{for} \quad T_2 < T \\ I_2\ (T_2 - T) + B & \text{for} \quad T_1 < T \leq T_2 \\ I_1\ (T_1 - T)^{2\beta} + I_2\ (T_2 - T) + B & \text{for} \quad T \leq T_1, \end{cases} \tag{9}$$

where $T_2 > T_1$ are the transitions temperatures, β is the critical exponent for the power law behavior and I_1, I_2 and B are constants. T_1 and T_2 are fitted globally whereas β, I_1, I_2 and B vary between data set. The obtained transition temperatures are $T_2 = 25.7(2)$ K and $T_1 = 20.8(1)$ K with the average value of $\beta = 0.32(3)$. Our data is generally well described with this model, see Fig. 1.

From the 8 magnetic Bragg peaks we can refine the magnetic structure at base temperature and obtain a combination of a C-type structure with moment components along a and b as well as a smaller A-type component with moments along c with $R_F = 0.132$. The ordered moment is $\mu = (2.23(2), 1.61(6), 0.33(3))\mu_B$ which yields an angle in the (a,b)-plane of $\varphi = 54(1)^\circ$. This is consistent with the angle of $\varphi \approx 60^\circ$ as obtained from the subsequent polarized neutron scattering experiment. In the polarized experiment we observed a possible change in crystal symmetry at T_1 and the results were inconclusive with respect to the existence of a spin component along c. At E5, we obtained a sizeable component along c with A-type symmetry like in the parent compound, $LiNiPO_4$ [8]. Our observations are therefore contradictory and inconclusive on this point.

The total ordered moment is $|\mu| = 2.76(3)\,\mu_{\rm B}$. For $LiFePO_4$ and $LiNiPO_4$ the measured ordered moments are respectively $4.2\,\mu_{\rm B}$ [**?**] and $2.2\,\mu_{\rm B}$ [9] per magnetic ion yielding an expected moment of $2.6\,\mu_{\rm B}$ for the chemical composition of the mixed sample. That is in very good agreement with the refinement result.

For the phase at intermediate temperatures, $T_1 \leq T \leq T_2$, it was not possible to unambigiously determine the magnetic structure from our E5 data. However, the results indicate a C-type structure with moments along b and $|\mu| = 1.27\,\mu_{\rm B}$ at 22 K.

4F1 experiment

In addition to data for the $(0,1,0)$ and $(0,0,1)$ magnetic Bragg peaks presented in the main text we also measured $(0,1,2)$ using polarized neutron diffraction at 4F1, see Figs. 2a-b. The SF intensity as collected in rocking scans

TABLE II. Results of the crystal structure refinements of $LiNi_{0.8}Fe_{0.2}PO_4$ from single-crystal neutron diffraction data collected at room temperature. The thermal parameters U_{ij} (given in $100\,\text{Å}^2$) are in the form $\exp\left[-2\pi^2\left(U_{11}H^2(a^*)^2+...+2U_{13}HL(a^*)(c^*)\right)\right]$, where H, K and L are Miller indices and a^*, b^* and c^* are the reciprocal lattice parameters. For symmetry reasons the values U_{12} and U_{23} of the atoms located at the Wyckoff position 4c are zero for $Pnma$. Site occupancies were refined for Li and O sites only. Within the errors only Li sites do not reach full occupancy.

Atom	Site	x	y	z	U_{11}	U_{22}	U_{33}	U_{12}	U_{13}	U_{23}	Occ.
Li	4a	0	0	0	1.75(19)	1.82(19)	1.20(14)	0.10(17)	-0.41(10)	-0.51(10)	0.94(3)
Fe	4c	0.27701(5)	1/4	0.98123(10)	0.36(2)	0.61(2)	0.57(2)	0	0.00(1)	0	0.2
Ni	4c	0.27701(5)	1/4	0.98123(10)	0.36(2)	0.61(2)	0.57(2)	0	0.00(1)	0	0.8
P	4c	0.09461(10)	1/4	0.41758(19)	0.34(4)	0.59(4)	0.37(3)	0	0.01(2)	0	1
O_1	4c	0.09928(9)	1/4	0.74208(17)	0.61(3)	0.96(4)	0.40(2)	0	0.00(2)	0	0.999(9)
O_2	4c	0.45275(9)	1/4	0.20116(18)	0.28(3)	0.92(4)	0.68(3)	0	0.00(2)	0	1.016(8)
O_3	8d	0.16571(6)	0.04314(12)	0.27867(13)	0.66(3)	0.75(3)	0.58(2)	0.17(2)	0.10(2)	0.03(2)	1.008(6)

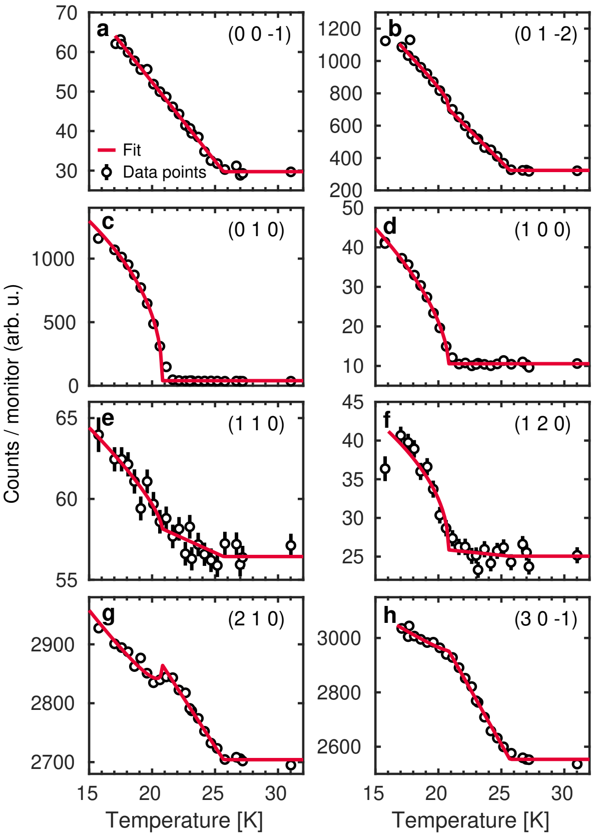

FIG. 1. **Neutron intensity as a function of temperature collected at E5.** Data points are shown with open black circles and the solid red lines are the global fit to Equation (9). The uncertainty of the neutron counts, N, follow Poisson counting statistics, $\sqrt{N}$. Two transitions are clearly observed.

for the three peaks are shown in panels d-f at selected temperatures. The Bragg peaks are resolution limited signifying long-range magnetic order in the system. The intensity ratio and overall resemblance of the data for $(0,0,1)$ and $(0,1,2)$ consolidate that the main magnetic structure component is C.

The angle, φ in the (a,b)-plane is calculated as:

$$\tan\varphi=\sqrt{\frac{I_a}{I_b}},\tag{10}$$

where $I_a = I_y^{\rm NSF} - I_x^{\rm NSF}$ or $I_a = I_x^{\rm SF} - I_y^{\rm SF}$ and $I_b = I_z^{\rm NSF} - I_x^{\rm NSF}$ or $I_b = I_x^{\rm SF} - I_z^{\rm SF}$. For $(0,1,2)$, I_b is corrected for the rotation between the sample coordinate system and the crystallographic axes, see Fig. 2c. Magnetic form factors and Lorentz factors may be omitted when regarding Bragg peaks individually like here. At the lowest probed temperatures we obtain canting angles respectively for $(0,1,2)$ and $(0,0,1)$ of $\varphi = 60.9(5)^\circ$ and $56.1(9)^\circ$.

Pyrocurrent measurements

The pyrocurrent in $LiNiPO_4$, $LiFePO_4$ and $LiNi_{0.8}Fe_{0.2}PO_4$ was measured using the quasi-static method [10]. The electric polarization, $P(T_1)$ at temperature T_1, was calculated from the measured pyrocurrent, I, using following formula:

$$P(T_1)=P(T_0)-\frac{1}{A}\int_{T_0}^{T_1} I(T)\left(\frac{dT}{dt}\right)^{-1}dT,\tag{11}$$

where $\frac{dT}{dt}$ is the temperature ramp rate and the minus sign in front of the integral is chosen such that $\Delta P > 0$ upon heating, as in our case. The constant, $P(T_0)$, is chosen such that the polarization is zero in the paramagnetic phase. The integration is performed numerically.

Since the polarization is calculated from the current, a small constant leak current in the measurement system will result in an overall slope in the polarization. This is relatively simple to correct for but any temperature dependent background signal may introduce features in the polarization that do not originate from the sample. In order to describe the background as accurately as possible, we construct a dataset based on the zero-field data and parts of the in-field data where it is known that there should be no signal, i.e. above the transition temperature. At zero field, the lithium orthophosphates do not support the ME effect. The background is different for

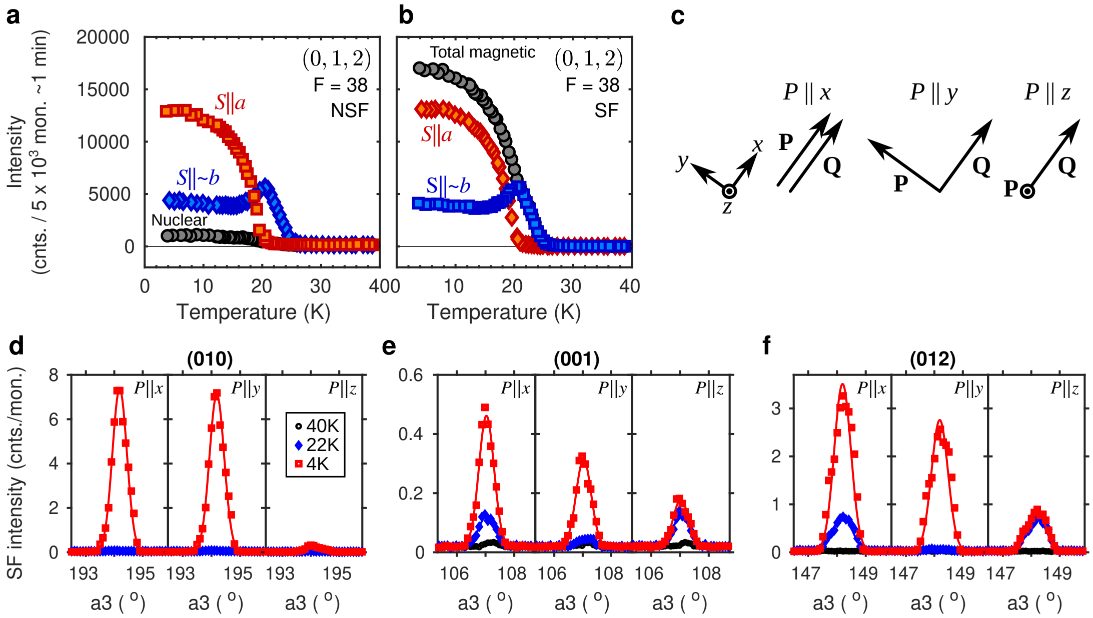

FIG. 2. **Polarized neutron diffraction data collected at 4F1.** The intensities as a function of temperature for $(0, 1, 2)$ in panels **a-b** clearly show two transitions. Circle, diamond and square symbols denote neutron polarization along x, y and z respectively. Nuclear and total magnetic signal are shown in black, spin components along a in red and spin components along b in blue. **c** The coordinate system used in the analysis of the polarized neutron diffraction data [5]. Panels **d-f** show rocking curves of the Bragg peaks respectively $(0, 1, 0)$, $(0, 0, 1)$ and $(0, 1, 2)$ at 40 (black circles), 22 (blue diamonds) and 2 K (red squares). Solid lines are Gaussian fits to the data. The uncertainty of the neutron counts, N, follow Poisson counting statistics, $\sqrt{N}$.

each electric and magnetic field direction. The pyrocurrent signal before and after background subtraction is shown in Fig. 3. It is clear that above the transition temperature we obtain a flat pyrocurrent signal.

The pyrocurrent is the derivative of the polarization and therefore this particular experimental method is well suited for characterizing second order phase transitions. First order phase transitions are identified by the step-like onset in the order parameter, i.e. in our case the electric polarization. Consequently the derivative at the transition is described by a delta function and is experimentally challenging to capture. Therefore, the pyrocurrent method is not a suitable choice for characterizing first order transitions such as for $LiNiPO_4$ [11]. On the other hand, it works well for second order transitions where the derivative of the order parameter is well-defined at all temperatures as is the case for $LiFePO_4$ [2] and $LiNi_{0.8}Fe_{0.2}PO_4$.

Figure 4 shows the electric polarization as obtained from Equation (11) for different magnetic field values. For $H||a$ and $H||b$, the ME curves are well defined but for $H||c$, the variations in the curves manifest a larger relative uncertainty. The anomalous behavior of the 1 T data is not understood. Linear fits to the average values of the polarization for $T < 5\,\mathrm{K}$ (see Fig. 3f in the main text) yield the ME coefficients listed in Table III. For completeness, the result for α_{bc} is included although here the pyrocurrent exhibits no spike as a function of temperature.

TABLE III. Magnetoelectric tensor coefficients for $LiNi_{0.8}Fe_{0.2}PO_4$ in units of $\mathrm{ps\,m^{-1}}$ as extracted from the linear fits to the mean polarization for $T < 5\,\mathrm{K}$ as a function of applied magnetic field (Fig. 3f in the main text). For comparison, the coefficient found using the same method for $LiFePO_4$ is $\alpha_{ab} = 0.6(1)\,\mathrm{ps\,m^{-1}}$.

α_{aa}	α_{ab}	α_{ac}	α_{ba}	α_{bb}	α_{bc}
2.3(6)	46(2)	2(3)	21(9)	25(4)	0(2)

Monte Carlo simulations

In the main part of the manuscript we use a simplified set of parameters for our Monte Carlo simulations but for completeness the full set of established magnetic exchange couplings and single ion anisotropies for both parent compounds, $LiNiPO_4$ and $LiFePO_4$, are given in Table IV. We performed additional simulations using the full parameter set but found qualitatively similar behavior. For those simulations we used the mean to describe inter-species couplings: $J^{\mathrm{Ni-Fe}} = \frac{1}{2}\left(J^{\mathrm{Ni}} + J^{\mathrm{Fe}}\right)$.

Susceptibility for $x = 0.06$

The main text of this paper is focused on intermediate ranges of x where $LiNi_{1-x}Fe_xPO_4$ hosts an oblique phase for $0.1 < x < 0.6$. The measured susceptibility for $x = 0.20$ is well reproduced by our Monte Carlo simulations, as seen by comparing Figs. 1c and 4a in the main

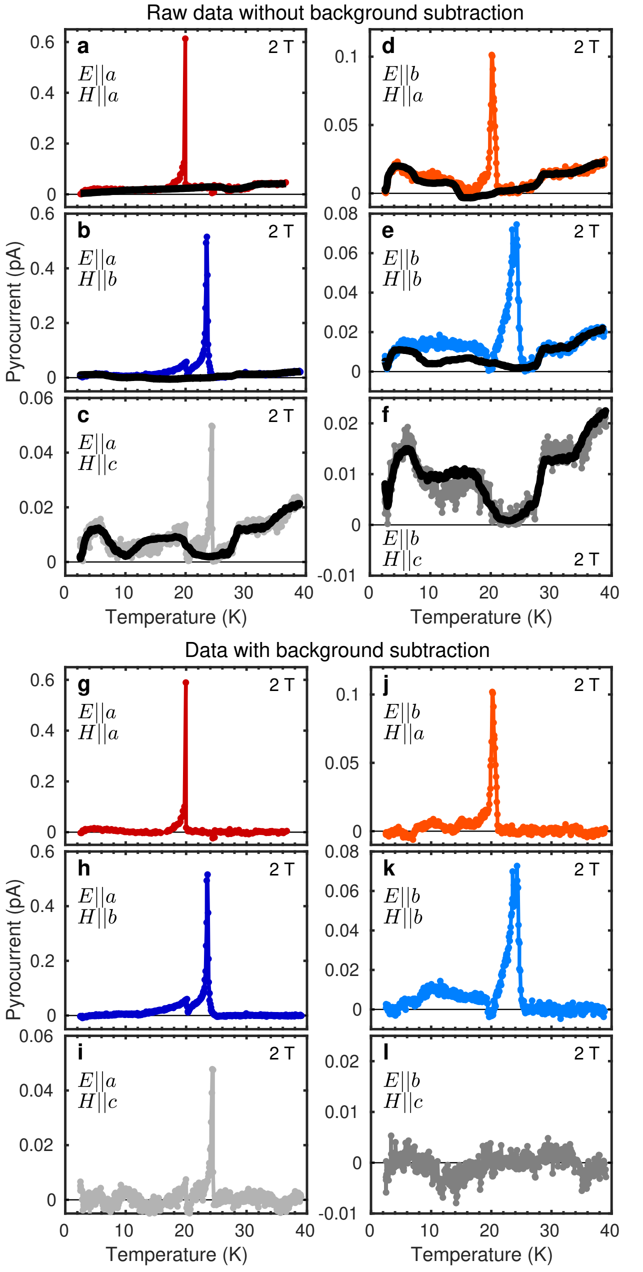

FIG. 3. **Background subtraction for the pyrocurrent measurements.** Top panels show the raw data (colored dots) with the background plotted on top (black curves). Bottom panels show the data after background subtraction. Only for $E||b$, $H||c$ there is no signal observed. The errors are in the order of 5 fA as seen by the fluctuations, e.g. in panel **l**.

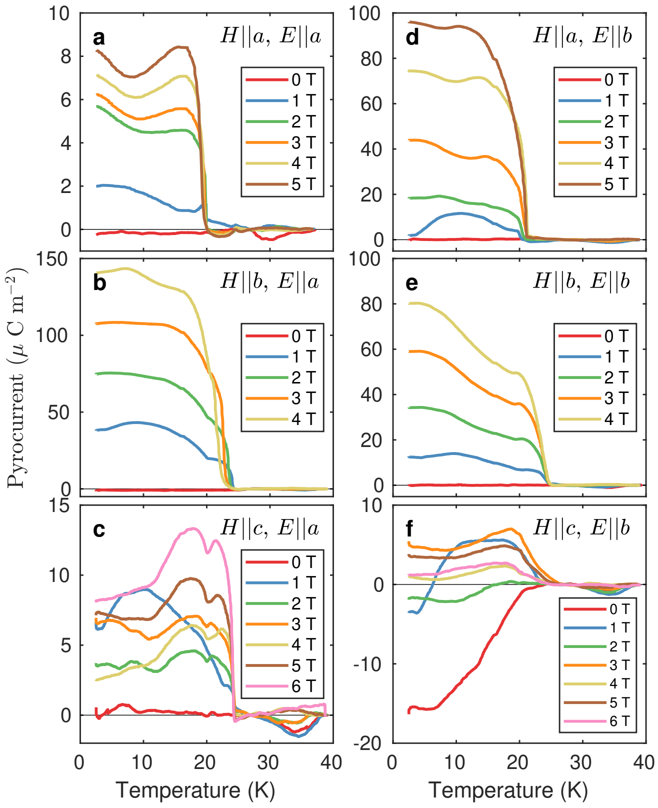

FIG. 4. **Electric polarization as a function of temperature at different magnetic field strengths.** Note the anomalous behavior at 1 T. The error on the polarization is estimated from the fluctuations in panel **f**. For the data at 2 T shown in Fig. 3d-e in the main text this yields uncertainties around $1\,\mu\mathrm{C\,m^{-2}}$

text. To further illustrate the success of these simulations we compare the measured and simulated susceptibility for $LiNi_{0.94}Fe_{0.06}PO_4$ in Fig. 5. The experiment was performed using a Cryogenic Ltd. cryogen free vibrating sample magnetometer and three separate crystals of masses 50, 40 and 20 mg. A magnetic field of 0.5 T was applied along the respective crystallographic axes. The simulated susceptibility was calculated from simulations performed without a Zeeman term in the Hamiltonian, as described in the Methods section.

The experimental susceptibility curves for $LiNi_{0.94}Fe_{0.06}PO_4$ with $H||a$ and $H||c$ are similar to those for $LiNiPO_4$ (compare Fig. 5a with Fig. 1d in the main text) indicating that the magnetic moments order along c as in the parent compound. For $H||b$, however, the susceptibility of $LiNi_{0.94}Fe_{0.06}PO_4$ displays a clear upturn below the transition temperature resembling a Curie tail associated with small amounts of paramagnetic impurities. This upturn is not present in $LiNiPO_4$.

TABLE IV. Exchange and single-ion anisotropy constants for $LiNiPO_4$ [12] and $LiFePO_4$ [2] given in milli electronvolt.

$LiNiPO_4$						
J_{bc}	J_b	J_c	J_{ab}	J_{ac}	D^a	D^b
1.04(6)	0.670(9)	-0.05(6)	0.30(6)	-0.11(3)	0.339(2)	1.82(3)
$LiFePO_4$						
J_{bc}	J_b	J_c	J_{ab}	J_{ac}	D^a	D^c
0.77(7)	0.30(6)	0.14(4)	0.14(2)	0.05(2)	0.62(12)	1.56(3)

The simulated susceptibility curves shown in Fig. 5b are in remarkably good agreement with the experimental data including the low-temperature increase for $H||b$. The easy axis for $LiFePO_4$ is b and locally this must also hold for the individual Fe^{2+} ions in $LiNi_{0.94}Fe_{0.06}PO_4$. We speculate that a small fraction of such ions act as magnetic impurities that are effectively decoupled from the surrounding antiferromagnetically ordered Ni^{2+} ions, and therefore behave as free magnetic ions upon applying a magnetic field along b.

Note that we do not observe the incommensurate phase reported in $LiNiPO_4$ [8] for small x in our simulations. However, our preliminary neutron diffraction study on $LiNi_{0.94}Fe_{0.06}PO_4$ shows that this phase persists and should be subject for future studies.

Local moment fluctuations

Both our experiments and simulations show that there is magnetic long range order in the $LiNi_{1-x}Fe_xPO_4$ system for all x. However, in the simulations we also see local fluctuations of the moment direction as illustrated in Fig. 6a. The site-dependent single-ion anisotropy means that the average direction of the moments vary depending on whether there is Ni or Fe on a specific site and which ion species reside on the neighboring sites, i.e. the local crystal field. In Fig. 6b-c we plot the spin components along a, b and c for the Ni and Fe sites individually. At small x, the Ni spins are ordered along c and for large x along b but with a crossover region with a large a-axis component. The picture is similar for the Fe spins except for small x where the spin component along b increases upon decreasing the Fe content. This is a result of the strong single-ion anisotropy of Fe and its large spin. Note that none of the spin components are zero due to the finite temperature in the simulations. A diffuse neutron scattering experiment would illuminate the local variation in moment orientation.

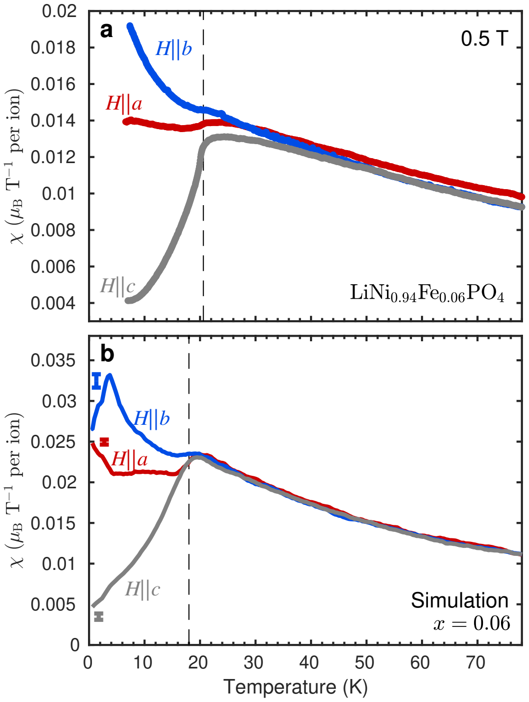

FIG. 5. **Susceptibility for $LiNi_{0.94}Fe_{0.06}PO_4$.** Magnetic susceptibility measured in an applied field of 0.5 T along a (red curve) b (blue curve) and c (grey curve) (**a**) and simulated for $x = 0.06$ (**b**). Transition temperatures are marked by vertical dashed lines. The largest errors estimated for the simulations are shown on the left in panel **b** and for the measurements the errors are smaller than the symbol size.

[1] I. Abrahams and K. S. Easson, "Structure of lithium nickel phosphate," *Acta Cryst. C* **49**, 925–926 (1993).
[2] R. Toft-Petersen, M. Reehuis, T. B. S. Jensen, N. H. Andersen, J. Li, M. D. Le, M. Laver, C. Niedermayer, B. Klemke, K. Lefmann, and D. Vaknin, "Anomalous magnetic structure and spin dynamics in magnetoelectric $LiFePO_4$," *Phys. Rev. B* **92**, 024404 (2015).
[3] R. P. Santoro, D. J. Segal, and R. E. Newnham, "Magnetic properties of $LiCoPO_4$ and $LiNiPO_4$," *J. Phys. Chem. Solids.* **27**, 1192–1193 (1966).
[4] R. P. Santoro and R. E. Newnham, "Antiferromagnetism in $LiFePO_4$," *Acta Cryst.* **22**, 344–347 (1967).
[5] R. M. Moon, T. Riste, and W. C. Koehler, "Polarization analysis of thermal neutron scattering," *Phys. Rev.* **181**, 920–931 (1969).
[6] J. R. Stewart, P. P. Deen, K. H. Andersen, H. Schober, J.-F. Barthélémy, J. M. Hillier, A. P. Murani, T. Hayesb, and B. Lindenau, "Disordered materials studied using neutron polarization analysis on the multi-detector spectrometer, D7," *J. Appl. Cryst.* **42**, 69–84 (2009).
[7] G. Ehlers, J. R. Stewart, A. R. Wildes, P. P. Deen, and K. H. Andersen, "Generalization of the classical xyz-polarization analysis technique to out-of-plane and inelastic scattering," *Rev. Sci. Instrum.* **84**, 093901 (2013).
[8] R. Toft-Petersen, J. Jensen, T. B. S. Jensen, N. H. Andersen, N. B. Christensen, C. Niedermayer, M. Kenzelmann, M. Skoulatos, M. D. Le, K. Lefmann, S. R. Hansen, J. Li, J. L. Zarestky, and D. Vaknin, "High-field magnetic phase transitions and spin excitations in magnetoelectric $LiNiPO_4$," *Phys. Rev. B* **84**, 054408 (2011).
[9] Thomas Bagger Stibius Jensen, Niels Bech Christensen, Michel Kenzelmann, Henrik Moodysson Rønnow, Christof Niedermayer, Niels Hessel Andersen, Kim Lefmann, Jürg Schefer, Martin v. Zimmermann, Jiying Li, Jerel L. Zarestky, and David Vaknin, "Field-induced magnetic phases and electric polarization in $LiNiPO_4$," *Phys. Rev. B* **79**, 092413 (2009).
[10] A. G. Chynoweth, "Dynamic method for measuring the pyroelectric effect with special reference to barium titanate," *J. Appl. Phys.* **27**, 78–84 (1956).
[11] D. Vaknin, J. L. Zarestky, J.-P. Rivera, and H. Schmid, "Commensurate-incommensurate magnetic phase transition in magnetoelectric single crystal $LiNiPO_4$," *Phys. Rev. Lett.* **92**, 207201 (2004).
[12] T. B. S. Jensen, N. B. Christensen, M. Kenzelmann,

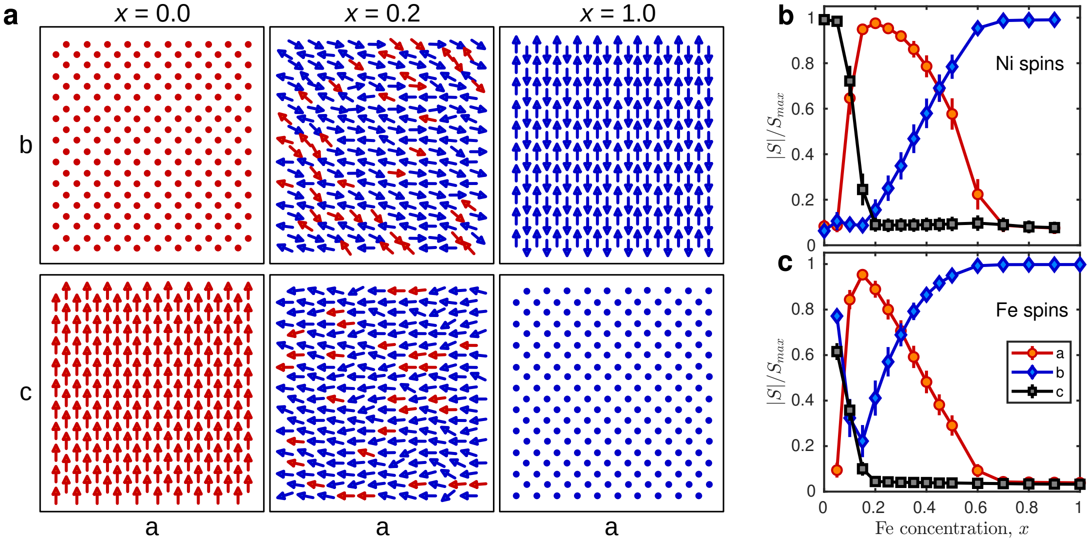

FIG. 6. **Fluctuation of local moments.** **a** Examples of system configurations for $x = 0.0, 0.2$ and 1.0 shown in the (a, b) and (a, c) planes (top and bottom row respectively). All moments are shown with the same length to clearly show their orientation. Red arrows correspond to Ni^{2+} and blue arrows to Fe^{2+}. In panels **b-c** are plots of the average of the absolute value of the spin components along the a (red circles), b (blue diamonds) and c (black squares) directions for Ni and Fe sites. The errors bars show the standard devitation.

H. M. Rønnow, C. Niedermayer, N. H. Andersen, K. Lefmann, M. Jiménez-Ruiz, F. Demmel, J. Li, J. L. Zarestky, and D. Vaknin, "Anomalous spin waves and the commensurate-incommensurate magnetic phase transition in $LiNiPO_4$," *Phys. Rev. B* **79**, 092413 (2009).
